# Molecular Simulations of Interface-Driven Crosslinked Network Formation and Mechanical Response in Composite Propellants

**DOI:** 10.3390/polym17131863

**Published:** 2025-07-03

**Authors:** Chen Ling, Xinke Zhang, Xin Li, Guozhu Mou, Xiang Guo, Bing Yuan, Kai Yang

**Affiliations:** 1 Center for Soft Condensed Matter Physics and Interdisciplinary Research & School of Physical Science and Technology, Soochow University, Suzhou 215006, China; 20244208009@stu.suda.edu.cn (C.L.); xkzhang@suda.edu.cn (X.Z.); 2 Hubei Institute of Aerospace Chemical Technology, Xiangyang 441003, China; 18638789202@163.com (X.L.); wdasdwfgfsg@163.com (G.M.); 3 Songshan Lake Materials Laboratory, Dongguan 523808, China; 4 Jiangsu Key Laboratory of Frontier Material Physics and Devices, Soochow University, Suzhou 215006, China

**Keywords:** composite solid propellants, two-step reaction, crosslinked network, interfacial defect, mechanical properties, molecular dynamics

## Abstract

Composite solid propellants, which serve as the core energetic materials in aerospace and military propulsion systems, necessitate tailored enhancement of their mechanical properties to ensure operational safety and stability. A critical challenge involves elucidating the interfacial interactions among the multiple propellant components (≥6 components, including the polymer binder HTPB, curing agent IPDI, oxidizer particles AP/Al, bonding agents MAPO/T313, plasticizer DOS, etc.) and their influence on crosslinked network formation. In this study, we propose an integrated computational framework that combines coarse-grained simulations with reactive force fields to investigate these complex interactions at the molecular level. Our approach successfully elucidates the two-step reaction mechanism propagating along the AP interface in multicomponent propellants, comprising interfacial self-polymerization of bonding agents followed by the participation of curing agents in crosslinked network formation. Furthermore, we assess the mechanical performance through tensile simulations, systematically investigating both defect formation near the interface and the influence of key parameters, including the self-polymerization time, HTPB chain length, and IPDI content. Our results indicate that the rational selection of parameters enables the optimization of mechanical properties (e.g., ~20% synchronous improvement in tensile modulus and strength, achieved by selecting a side-chain ratio of 20%, a DOS molar ratio of 2.5%, a MAPO:T313 ratio of 1:2, a self-polymerization MAPO time of 260 ns, etc.). Overall, this study provides molecular-level insights into the structure–property relationships of composite propellants and offers a valuable computational framework for guided formulation optimization in propellant manufacturing.

## 1. Introduction

Composite solid propellants (CSPs) serve as the primary energetic materials in modern aerospace and military propulsion systems due to their exceptional energy density, tunable combustion characteristics, and superior performance [1,2,3,4]. These materials are highly filled elastomeric composites, typically composed of a polymeric binder (fuel component) and a majority of solid fillers, such as oxidizer particles and aluminum powder, uniformly dispersed within the matrix. However, the extreme manufacturing and working conditions impose stringent mechanical demands on CSPs, requiring them to withstand thermal and pressure loads while resisting aging-induced degradation [5,6,7]. Additionally, CSPs must endure complex mechanical stresses arising from shocks, vibrations, and pressure fluctuations. Under such demanding conditions, tensile and shear stresses may induce microscopic defects within CSPs, potentially leading to significant performance degradation or even catastrophic failure [8,9,10]. Therefore, a fundamental understanding of the microscopic structures of CSPs at the molecular level and their correlation with mechanical properties is critical for ensuring the safety and reliability of propulsion systems [11,12].

Usually, CSPs contain many propellant components, such as a polymer binder (hydroxyl-terminated polybutadiene, HTPB), solid oxidizer (Ammonium perchlorate, AP), plasticizer (Dioctyl sebacate, DOS), curing agent (Isophorone diisocyanate, IPDI), and bonding agent (Tris-1-(2-methylaziridinyl) phosphine oxide, MAPO, Boron trifluoride triethanolamine complex, T313), etc. Thus, the formulation selection of polymer binders and additives is crucial for controlling the mechanical performance of CSPs [13,14,15,16,17,18]. The polymeric binders not only provide structural integrity due to its high content and molecular weight but also ensure strong interfacial adhesion with oxidizer particles and other functional additives. The HTPB binder undergoes curing reactions with isocyanate groups, forming a polyurethane elastomer that exhibits optimal stiffness, high elongation at break, excellent thermal stability, and superior water resistance. Bonding agents, such as aziridines and their derivatives (e.g., MAPO), as shown by previous studies [19,20], can adsorb onto AP surfaces through hydrogen bonding and undergo ring-opening polymerization, forming an interfacial layer with high modulus and tear resistance. Furthermore, these bonding agents react with isocyanate-based curing agents, significantly strengthening the interface between AP particles and the polymer matrix. These synergistic reactions contribute to an integrated crosslinked network, enhancing the overall mechanical robustness of CSPs [1,20,21]. On the other hand, interfacial defects—particularly debonding under tensile loading—are among the primary causes of mechanical property degradation in CSPs. Such defects often arise from voids near the interface due to spatial heterogeneity, leading to crack initiation and propagation [14,21,22]. Collectively, these intricate microscopic factors, primarily governed by interfacial interactions and reactions between polymer binders and additives, play a crucial role in determining the mechanical behavior of CSPs [23,24,25,26]. Nevertheless, a comprehensive molecular-level understanding of interfacial properties and their influence on mechanical performance remains a significant challenge [8,27,28].

In this work, an integrated computational framework combining coarse-grained molecular dynamics (CGMD) simulations and reactive force field methods is developed to elucidate the microscopic mechanisms governing the mechanical properties of multicomponent CSPs. This framework reproduces the sequential two-step reaction process during CSP production well: (1) surface-catalyzed self-polymerization of the bonding agent MAPO on AP particles, followed by (2) diverse crosslinking reactions mediated by curing agents. Moreover, through tensile simulations, we find that microscopic defects preferentially nucleate at interfacial regions, while the conformational changes of HTPB chains and interfacial interactions collectively determine the system’s mechanical response. In addition, based on these results, we demonstrate that CSP formulations can be further optimized to achieve an approximately 20% simultaneous improvement in both elastic modulus and maximum tensile strength by properly changing critical factors, such as the self-polymerization time, chain length and side-chain ratio of HTPB, IPDI content, etc. Our study provides molecular-level insights into the interfacial interactions within CSPs and the formation mechanisms of crosslinked networks and offers fundamental guidance for designing propellants with enhanced mechanical performance.

## 2. Materials and Methods

### 2.1. Coarse-Grained Models for Simulations

**Figure 1:**
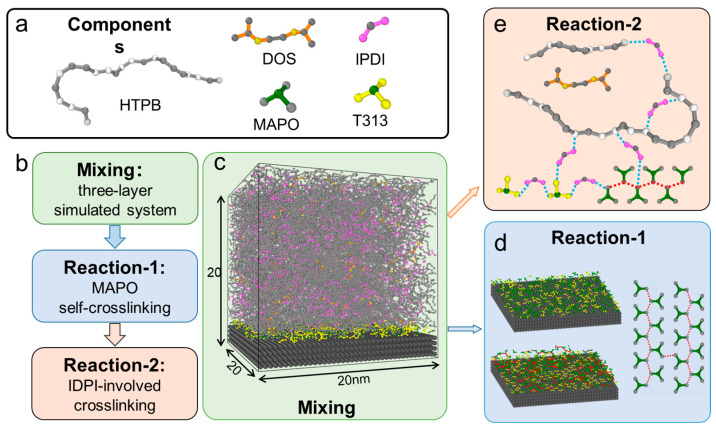
Simulation models and framework for CSP. (**a**) Coarse-grained representations of CSP components. (**b**) Workflow illustrating the crosslinking simulation process. (**c**) The three-layer simulation system, designed to replicate experimental conditions, comprises (from top to bottom) (i) the polymer blend layer (HTPB + DOS + IPDI), (ii) the bonding layer (T313 + MAPO), and (iii) the solid particle layer (AP). The crosslinking network formation involves two key reactions: (**d**) Reaction-1: self-polymerization of MAPO at the AP interface (newly formed bonds indicated by red dashed lines), followed by (**e**) Reaction-2: IPDI-mediated crosslinking (blue dashed lines), which forms covalent bonds with (i) HTPB (terminal hydroxyl groups and vinyl-1,2 side chains), (ii) ring-opened MAPO, and (iii) T313 molecules.

All MD simulations were performed using the Galamost software package version 1.40, (Institute of Chemistry, Chinese Academy of Sciences, Beijing, China), a GPU-accelerated program designed for efficient coarse-grained molecular dynamics simulations, under isothermal–isobaric (NPT) ensemble conditions [29]. The Martyna–Tobias–Klein (MTK) barostat and Nosé–Hoover thermostat were utilized to maintain a constant system pressure of 1 bar and temperature of 300 K [30]. The selected temperature corresponds to typical experimental sample preparation and storage conditions (i.e., room temperature). However, we note that temperature could significantly affect the mechanical properties of analogous material systems [8,19]. Short-range interactions were modeled using the LJCoulombShiftForce module, which ensures a smooth decay to zero at the cutoff distance. Figure 1a illustrates the coarse-grained molecular representations of the simulated system components, including the polymer binder HTPB, curing agent IPDI, bonding agents MAPO/T313, and plasticizer DOS (note that oxidizer particle AP is shown in Figure 1c). The all-atom-to-coarse-grained mapping protocols and corresponding force field parameters are provided in detail in Figure S1 and Table S1. Specifically, the HTPB chains were initially constructed with a polymerization degree of 50, where chain length, functionality, and side-chain reactivity were implemented as adjustable parameters for subsequent investigations into mechanical properties. The IPDI molecule was coarse-grained into a three-bead model with asymmetric arm lengths to reflect the differential reactivity of its benzene ring termini [31]. Both MAPO and T313 were represented by four-bead models with enhanced interaction potentials to account for their strong hydrogen-bonding characteristics with solid particles. Due to the relatively large size of the AP particle, it was simplified as a solid surface in our simulations. Specially, the AP surface was constructed by arranging CG beads along the (001) crystal orientation, with a surface thickness of 2 nm.

In addition, in our work, the component molecules were basically modeled using standard Martini bead types (e.g., P1, P3, C3, C5, and Q0) [32,33,34,35,36]. Moreover, due to the requirements of the reactive force field (which necessitates distinct naming conventions for different molecule types), certain bead designations were modified: P1 beads were re-named as IP or HP1, and C3 beads were uniformly designated as CR (see Table S1). Given the system’s specificity, some molecular structures could only be approximated using Martini beads with similar properties. This approximation might potentially affect component density distributions. To compensate, we proportionally scaled up the σ values for all bead pairs while specifically strengthening the P3-Q0 interactions (unique to the bonding agent and AP, respectively) to preserve experimental density fidelity (see Figure S2). Note that the extreme complexity of our multicomponent system made it challenging to achieve simultaneous convergence for all molecules by using classical coarse-graining approaches. But the emerging machine learning approaches (e.g., machine learning-informed energy renormalization) may be promise for addressing such challenges to pursue more accurate parameterization schemes [37,38].

### 2.2. Simulations System Setup and Workflow

Based on experimental observations [20], the simulation workflow consists of three key stages (Figure 1b): (1) component mixing, (2) MAPO self-polymerization (Reaction-1), and (3) IPDI-involved network crosslinking reactions (Reaction-2). Experimental evidence suggests that the bonding agents MAPO and T313 can ultimately coat the AP surface and undergo self-crosslinking under appropriate conditions to form a tear-resistant layer, though this process requires prolonged mixing durations. Accordingly, in our simulation protocol, we directly established a three-layer composite model with systematically arranged components as the initial mixing configuration, followed by 100 ns NPT equilibrium simulations of the component mixtures. Figure 1c (visualized using OVITO software, version 2.9.0 [39]) (OVITO GmbH, Darmstadt, Germany) illustrates this hierarchical structure, consisting of (1) a blended binder layer (top) containing IPDI and HTPB for crosslinked network formation, with uniformly dispersed DOS plasticizer; (2) an interfacial reaction layer of MAPO and T313, which serves as the site for subsequent MAPO self-polymerization; and (3) a rigid solid particle layer (bottom).

The subsequent stages involve the formation of a crosslinked network through a two-step reaction process (Figure 1d,e). The first step (Reaction-1, simulation time varies from 20 ns to 340 ns) corresponds to MAPO self-polymerization, where reactions occur between arms of different MAPO molecules (see Figure 1c). Based on experimental observations [20,40], these reactions are spatially confined to the AP interface via a constraint algorithm that deactivates reactive sites beyond a critical distance (~1 nm) from AP particles. The second step (Reaction-2, 600 ns simulations) involves IPDI-mediated network crosslinking through reactions between IPDI, HTPB, MAPO, and T313, ultimately yielding a fully integrated network structure (Figure 1d). Following MAPO polymerization, IPDI initiates reactions with HTPB terminal hydroxyl groups, T313 functional groups, and ring-opened MAPO arms, forming a dense, three-dimensional network. Moreover, in addition to the primary hydroxyl-terminated reactions, the vinyl-1,2 side-chain isomer of HTPB—containing reactive double bonds—also participates in crosslinking, further increasing network connectivity. Notably, previous studies have demonstrated that such side-group incorporation substantially influences both HTPB’s properties and the resulting polyurethane’s mechanical characteristics, including its tensile strength, elongation at break, and hardness [41]. In addition, it should be noted that MAPO participates in both reactions, whereas T313 is only involved in Reaction-2 through its interaction with the curing agent IPDI.

In our simulations, these reactions are modeled using the stochastic polymerization and reaction algorithm developed by Liu et al. [42,43,44]. In this approach, an active center reacts with nearby monomers within a defined reaction radius with probability Pr; once the reaction event is achieved, the active center transfers to the newly incorporated monomer, while the original center becomes deactivated. This “active center transfer” mechanism governs the polymerization process, with each active site permitted to participate in only one reaction event to prevent multiple reactions. In our cases, for optimal simulation efficiency while preserving physical accuracy, all reaction probabilities are standardized at 2%, except for the vinyl-1,2 side reaction, which was set to 0.002% to reflect its high activation energy barrier [45,46].

### 2.3. Mechanical Characterization Through Tensile Simulations

**Figure 2:**
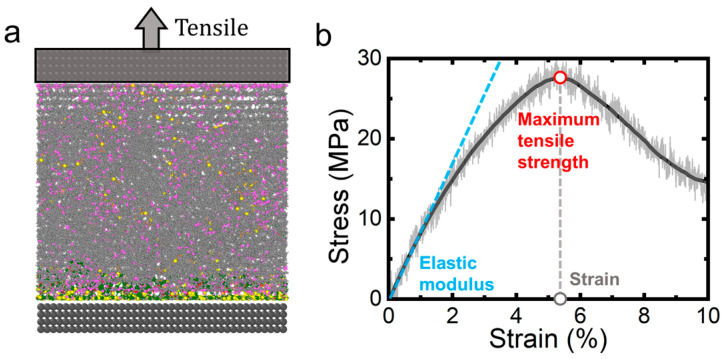
Tensile simulation and mechanical property measurement. (**a**) Uniaxial tension is applied along the *z*-axis to simulate the tensile process. (**b**) Characteristic stress–strain curve obtained from tensile simulation, with key mechanical parameters identified through curve fitting, including strain with maximum tensile strength, maximum tensile strength (peak point stress), and elastic modulus (slope of blue dashed line).

Uniaxial tensile simulations are conducted along the *z*-axis (normal to the solid surface) under NVT ensemble conditions. As shown in Figure 2a, the equilibrated crosslinked system was subjected to a constant strain rate of 5 × 10^−7^ s^−1^, allowing for sufficient simulation time for complete elastoplastic deformation and defect formation.

The mechanical response is characterized by monitoring the stress–strain evolution (Figure 2b), from which three key parameters are derived through curve fitting, including the linear strain, maximum tensile strength, and the corresponding strain. Notably, the simulation protocol excludes chemical bond rupture events. System size independence is verified through scale-up tests (Figure S3), demonstrating consistent mechanical properties across different simulation dimensions.

## 3. Results and Discussion

**Figure 3:**
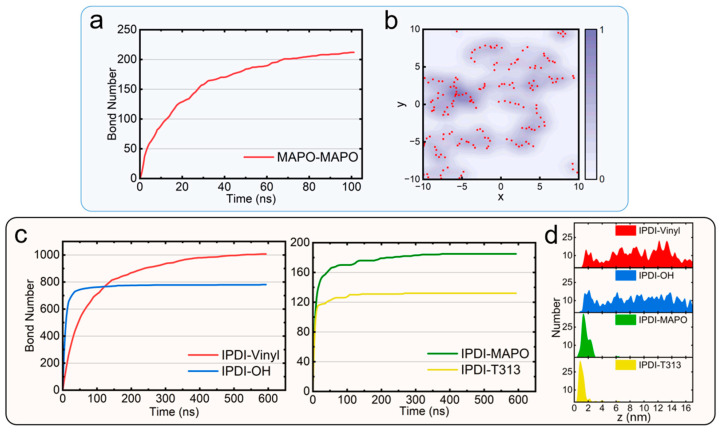
Reaction kinetics and bond formation analysis. (**a**) Temporal evolution of bond formation during MAPO self-polymerization (Reaction-1). (**b**) Two-dimensional spatial distribution of MAPO-derived bonds at the AP interface (z = 0), where purple shading indicates bonding probability (intensity correlates with probability), and red dots denote bonding sites. (**c**) Reaction kinetics of IPDI-mediated crosslinking (Reaction-2) with HTPB (through both vinyl and terminal hydroxyl groups), T313, and ring-opened MAPO. (**d**) Spatial distribution along the z-direction of different IPDI-mediated bonds near the AP surface (z = 0).

Our simulation approach provides a molecular-scale resolution of the CSP production process, enabling fundamental understanding of performance-determining mechanisms for targeted mechanical optimization. The formation of crosslinked networks through the two-step reactions is the core of CSP production. In Reaction-1 (MAPO self-polymerization), the temporal evolution of bond formation follows a power-law growth trend, with decreasing monomer concentration leading to progressively slower bond formation kinetics (Figure 3a). This reaction primarily occurs near the AP interface. Figure 3b shows the 2D spatial distribution of MAPO bonding positions, revealing non-uniform patterns where MAPO self-polymerization occurs preferentially in specific localized regions (indicated by purple and red dots in Figure 3b). These reactive zones are interspersed with areas containing T313 and partially unreacted MAPO (white regions in Figure 3b). Note that such irregular distributions of MAPO bonding positions are commonly observed across different systems (Figure S4), probably arising from the stochastic nature of the single-armed polymerization reaction. This heterogeneous spatial arrangement may create localized stress differences that could further initiate mechanical failure during tensile deformation. Notably, while the reaction probability affects the rate of MAPO self-polymerization, it ultimately leads to less than 10% variation in the total number of bonds formed after sufficient reaction time (Figure S5). In Reaction-2, IPDI reacts with HTPB, MAPO, and T313 to form an integrated network structure. Figure 3c reveals that the bond formation kinetics still follow a power-law trend, with most reactions reaching plateaus rapidly (<30 ns). Notably, the IPDI-vinyl (HTPB) reaction, though slower (saturating within 200 ns), ultimately forms more bonds than the IPDI–OH (HTPB) reaction. A critical finding is IPDI’s role as a mobile crosslinker: it diffuses through the upper layer to participate in all secondary reactions, as evidenced by the z-directional distribution of IPDI bonding sites (Figure 3d). The dominant reaction peak occurs near the AP surface (around z = 1.5 nm), corresponding to interfacial crosslinking between IPDI and the pre-formed 2D MAPO/T313 network. This interfacial reaction zone is directly linked to the defect formation mechanisms, as discussed in the subsequent section.

**Figure 4:**
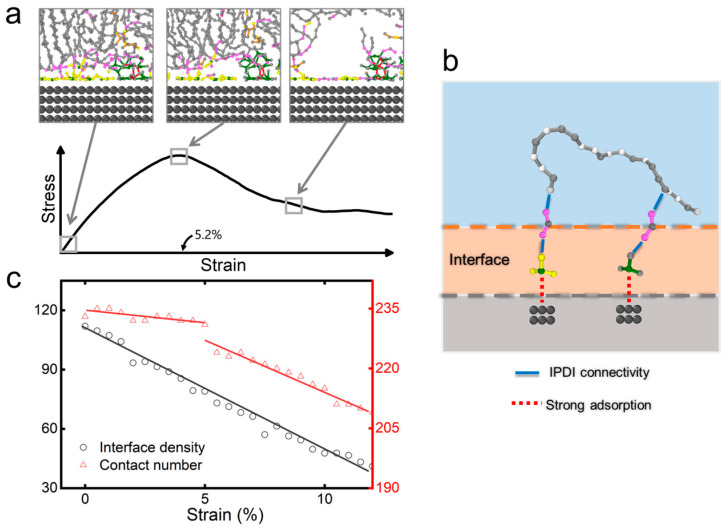
Defect formation under tensile simulation. (**a**) Sequential snapshots of interfacial morphology at increasing strain levels, highlighting defect nucleation preferentially at interfacial regions. (**b**) Schematic representation of the interfacial defect formation. (**c**) Strain-dependent evolution of (i) interfacial (mass) density and (ii) contact number between bonding agents and AP particles.

Following crosslinked network formation, we conduct tensile simulations to evaluate the system’s mechanical response (Figure 2). Especially, defects nucleate at sufficiently large strains, ultimately leading to material rupture. As shown in Figure 4a and Figure S6, defect initiation occurs preferentially at the AP particle–polymer interface, corresponding to a characteristic stress drop in the stress–strain profile (Figure 4a). Our analysis reveals a two-stage defect evolution process mediated by interfacial interactions: (1) initial bonding agent (MAPO/T313)-AP contact maintenance through strong adsorption (red dashed line, Figure 4b), followed by (2) interfacial debonding. Figure 4c quantifies this process through two complementary metrics: (i) the mass density near the AP interface shows a linear decrease with strain, while (ii) the bonding agent–AP contact number exhibits a biphasic behavior—a gradual initial decline followed by rapid decrease beyond a critical strain threshold (~5%)—leading to rapid crack propagation. Notably, this transition point coincides precisely with the maximum tensile strength in Figure 4a, indicating the onset of catastrophic interfacial failure (Figure S7). This hierarchical failure mechanism indicates that pre-existing interfacial defects from the crosslinking step may influence the composite’s ultimate mechanical performance.

**Figure 5:**
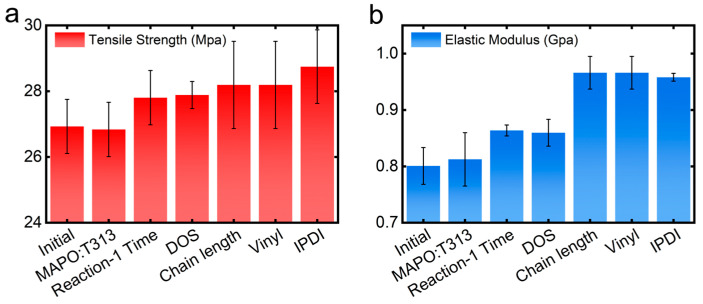
Dependence of mechanical properties on formulation parameters: (**a**) tensile strength and (**b**) elastic modulus as functions of key compositional variables. Error analysis is based on three independent replica simulations.

Our computational framework, which integrates the atomic-resolution capabilities of MD with a parametrically tunable propellant model, provides a way to explore the CSP formulation space for optimizing mechanical properties, including the tensile strength and elastic modulus. Herein, we mainly focus on three classes of molecular-level design parameters: (1) the self-polymerization duration in Reaction-1 governing reaction kinetics, (2) HTPB chain length and side-chain vinyl group reactivity associated with polymer architecture, and (3) compositional factors such as MAPO/T313 ratio and DOS/IPDI numbers. Notably, optimizing these parameters is a non-trivial task due to their complex influence on mechanical properties. As a benchmark approach, we herein employ a one-factor-at-a-time optimization strategy, mirroring experimental protocols by varying individual parameters step by step while maintaining others at baseline values. The optimal value for each parameter is determined by either maximizing key mechanical properties or achieving an optimal balance among them (see Figure S8). Figure 5 illustrates the performance enhancements achieved relative to the initial formulation. Our analysis reveals Reaction-1 duration and IPDI content as primary determinants of the tensile strength, whereas the elastic modulus shows strongest dependence on the HTPB chain length. Generally, these three parameters affect the molecular architecture of the polymer binder and the critical interfacial interactions within the composite system.

**Figure 6:**
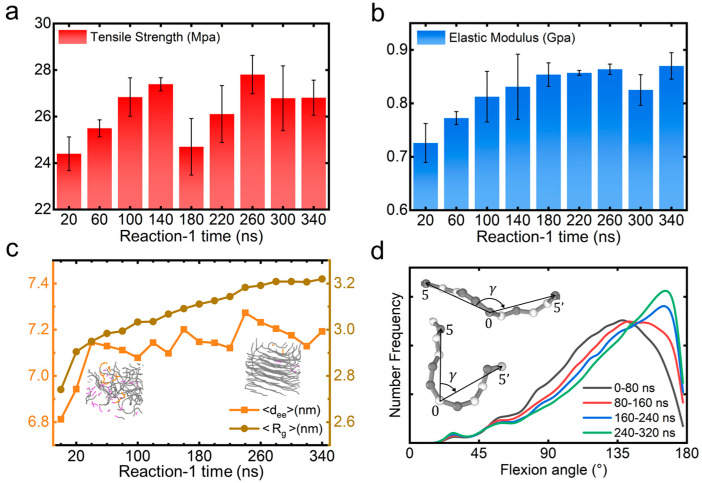
Influence of Reaction-1 self-polymerization time on mechanical and structural properties: (**a**) tensile strength, (**b**) elastic modulus evolution, (**c**) conformational changes characterized by d_ee_ and R_g_, and (**d**) the average flexion angle of HTPB for different time periods (every 80 ns). Herein, the flexion angle is the interior angle between two vectors from the target particle 0 to the particles 5 and 5′. Error analysis is based on three independent replica simulations.

As a representative case, we analyze the impact of the MAPO self-polymerization duration in Reaction-1 on the mechanical properties. Figure 6a,b demonstrate the changes of tensile strength and elastic modulus with this duration. Below 140 ns, both properties increase monotonically, correlating with progressive MAPO network formation (as shown in Figure 3a). However, beyond this time point, distinct behaviors emerge: the tensile strength exhibits non-monotonic variations (characterized by rapid decline followed by partial recovery), while the elastic modulus continues increasing until reaching 260 ns. The optimal balance between these two properties occurs at 260 ns, where these competing trends reach an advantageous equilibrium.

Furthermore, the MAPO self-polymerization duration significantly influences HTPB chain conformation, as evidenced by the evolution of the mean end-to-end distance (⟨d_ee_⟩) and radius of gyration (⟨R_g_⟩), shown in Figure 6c. It is found that ⟨d_ee_⟩ initially increases linearly before entering an oscillatory regime, while ⟨R_g_⟩ exhibits monotonic growth until reaching a plateau at ~300 ns. The optimal 260 ns time point coincides with maximum ⟨d_ee_⟩ and elevated ⟨R_g_⟩, indicating enhanced HTPB chain extension and spatial expansion that contribute to improved mechanical performance. Moreover, we observe a structural transition in HTPB from amorphous entanglement to quasi-crystalline alignment with parallel chain orientation during MAPO self-polymerization (Figure 6c insets) [26,47,48]. This transition is further supported by flexion angle distributions (Figure 6d), where the characteristic angle (defined in Figure 6d inset) [49] increases substantially with polymerization time. However, this crystallization phenomenon presents a mechanical paradox: while improving stiffness metrics, it simultaneously reduces material ductility, as evidenced by decreased elongation at break (Figure S8). The competing effects of conformational ordering (enhancing rigidity) and crystallinity (causing embrittlement) highlight the critical need for balanced parameter optimization in propellant design.

**Figure 7:**
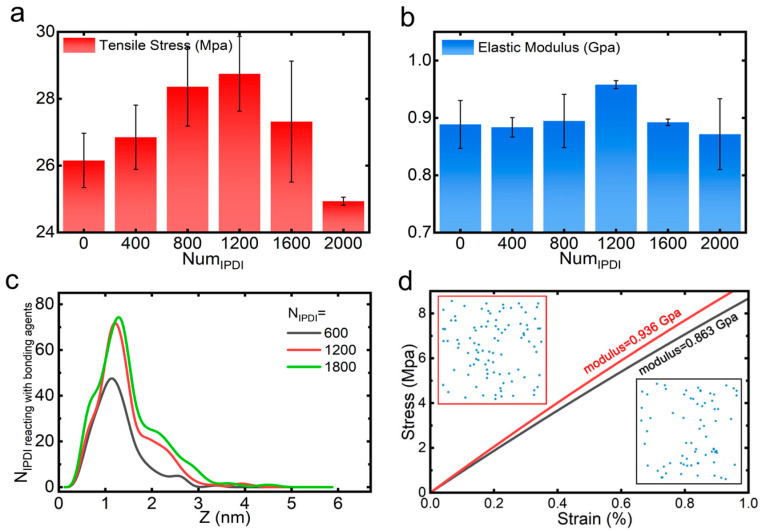
IPDI concentration dependence of (**a**) tensile strength and (**b**) elastic modulus. (**c**) *Z*-axis distribution of IPDI-mediated bonds with bonding agents. (**d**) Linear elastic stress–strain behavior for systems containing 800 (black) and 1200 (red) IPDI molecules, with insets showing the 2D spatial distribution of effective IPDI bridging connections between binder and bonding agent layers. Error analysis is based on three independent replica simulations.

In addition, as a key crosslinking agent, IPDI actively participates in network formation. Therefore, the effect of IPDI number on the mechanical properties is also analyzed. Our analysis reveals its non-monotonic concentration-dependent effects on mechanical properties (Figure 7a,b): both the tensile strength and elastic modulus initially increase with IPDI content, peak at N = 1200, then undergo decline with further addition. This behavior correlates directly with interfacial enhancement at the AP particle–polymer interface. As illustrated in Figure 7c, interfacial reactions between IPDI and bonding agents intensify with increasing IPDI content until reaching saturation at N = 1200. Higher IPDI concentrations improve connectivity between the binder and coupling agent layers, thereby enhancing the elastic modulus. Figure 7d compares stress–strain curves in the linear elastic region for systems with N = 800 and 1200. The inset demonstrates that a higher IPDI content (N = 1200) leads to a denser and more uniform distribution of reactive IPDI, which strengthens interfacial resistance to tensile deformation under strain. All these results suggest that optimal mechanical performance requires balanced IPDI content to maximize interfacial connectivity.

In general, our simulations reveal that while individual compositional variables modulate specific aspects of the crosslinked network, their synergistic interactions govern the emergent structural properties, including network topology, connectivity, and stiffness. Critical formulation parameters—particularly MAPO self-polymerization duration, HTPB chain architecture, and IPDI stoichiometry—collectively determine the system’s mechanical performance and load-bearing capacity. Especially, using the optimized parameters such as referred in Figure 6a,b and Figure 7a,b, we develop a hierarchical parameter optimization protocol to systematically improve mechanical performance. While competing effects among intrinsic factors, rational parameter selection nevertheless achieves significant enhancements, as demonstrated in Figure 5. Our optimized formulation (MAPO: T313 ratio = 1:2, MAPO self-polymerization time = 260 ns, DOS molar ratio = 2.5%, HTPB chain length = 30 monomers, vinyl side-chain ratio = 20%, and IPDI molar ratio = 59%) yields simultaneous 20% improvements in both the tensile modulus and strength relative to baseline systems (Figure 5). It is also worth noting that formulation optimization presents a significant challenge for CSPs. Herein, we only demonstrate a preliminary application of our integrated computational framework for quantitatively analyzing the effects of key parameters on mechanical properties that are difficult to characterize experimentally. In the future, the implementation of advanced optimization strategies, such as genetic algorithms, Bayesian optimization, and machine learning approaches, could offer a promising pathway for efficient identification of optimal CSP formulations [50,51].

## 4. Conclusions

In summary, we have developed an integrated computational framework combining coarse-grained molecular dynamics simulations with reactive force field methods to elucidate the mechanisms of self-polymerization and crosslinking network formation in CSPs. This approach successfully reproduces the two-step reaction process and characterizes the mechanical response under strain conditions. Most importantly, we uncover fundamental structure–property relationships that govern the performance of CSPs. For instance, we demonstrate that the duration of MAPO self-polymerization is a critical control parameter, with an optimal duration of approximately 260 ns enhancing HTPB chain extension while promoting favorable molecular ordering for synergistic mechanical improvement. Additionally, the IPDI content exhibits a non-monotonic influence on mechanical properties, achieving a peak at N = 1200 due to optimal interfacial bridging effects. Through systematic optimization of parameters—including the MAPO/T313 ratio (1:2), DOS content (2.5%), HTPB chain length (30 monomers), vinyl side-chain ratio (20%), and IPDI molar ratio (59%)—we achieve a concurrent 20% enhancement in key mechanical properties compared to conventional formulations. Overall, the developed framework provides unparalleled molecular-level insights into CSP structure–property relationships and establishes a paradigm for the rational design of high-performance propellants through computationally guided formulation optimization.

## Data Availability

Data are contained within the article.

## Supplementary Materials

The following supporting information can be downloaded at: https://www.mdpi.com/article/10.3390/polym17131863/s1, Table S1: Setting of LJs, bonds, and angles parameters in our model; Figure S1: Molecular structures and corresponding coarse-grained modeling scheme; Figure S2. Density distribution of the simulated system, consistent with experimental data and prior all-atom simulations; Figure S3: The mechanical properties in initial and scale-up systems; Figure S4. Two-dimensional spatial distribution of MAPO-derived bonds at the AP interface at different MAPO:T313 ratios; Figure S5. The bond formation process of MAPO self-polymerization with different Pr; Figure S6: Snapshots of the system under different strains; Figure S7: From left to right: (1) the bonding agent is adsorbed onto the AP surface, (2) the bonding agent begins to detach from the interface, and (3) the bonding agent completely departs from the interface; Figure S8: Strain at maximum tensile strength, maximum tensile strength, and elastic modulus as functions of (a) the MAPO:T313 ratio, (b) the duration of the first-step reaction, (c) the amount of DOS, (d) the HTPB chain length, (e) the proportion of vinyl side chains, and (f) the amount of IPDI. (g) Evolution of mechanical properties during the optimization process.

## References

[1] Yadav A., Pant C.S., Das S. (2020). Research Advances in Bonding Agents for Composite Propellants. Propell. Explos. Pyrot..

[2] Yadav N., Srivastava P.K., Varma M. (2021). Recent Advances in Catalytic Combustion of AP-Based Composite Solid Propellants. Def. Technol..

[3] Galavotti A., Noè C., Polizzi G., Antonaci P., Maggi F., Masseni F., Pastrone D. (2023). Solid Rocket Propellant Photo-Polymerization with an In-House LED-UV Prototype. Polymers.

[4] Hua C., Ye B., Yin S., Qiu M., Wang J., An C. (2024). Interfacial Engineering Endowing Ammonium Perchlorate with High Mechanical Properties and Energy-Release Efficiency. Ceram. Int..

[5] Zhang H., Liu M., Miao Y., Wang H., Chen T., Fan X., Chang H. (2020). Dynamic Mechanical Response and Damage Mechanism of HTPB Propellant under Impact Loading. Materials.

[6] Wu Y., Fan Y., Chen X., Wen J., Wang Q., Huang J. (2023). Low/Intermediate Speed Impact-Induced Ignition and Damage of a Novel High-Energy Solid Propellant. Propell. Explos. Pyrot..

[7] Yıldırım H.C., Özüpek Ş. (2011). Structural Assessment of a Solid Propellant Rocket Motor: Effects of Aging and Damage. Aerosp. Sci. Technol..

[8] Liu Y., He J., Xian W., Li Y. (2023). Impact Velocity and Temperature Effects on the Shock Wave Propagation and Spallation of Hydroxyl-Terminated Polybutadiene: A Molecular Dynamics Study. ACS Appl. Polym. Mater..

[9] Yin S., Lu Z., Bai H., Liu X., Li H., Hu Y. (2022). Functionalized GO/Hydroxy-Terminated Polybutadiene Composites with High Anti-Migration and Ablation Resistance Performance. Polymers.

[10] Zhang Y., Tian Y., Zhang Y., Fu X., Li H., Lu Z., Zhang T., Hu Y. (2022). Improvement in Migration Resistance of Hydroxyl-Terminated Polybutadiene (HTPB) Liners by Using Graphene Barriers. Polymers.

[11] Marimuthu R., Rao B.N. (2013). Development of Efficient Finite Elements for Structural Integrity Analysis of Solid Rocket Motor Propellant Grains. Int. J. Pres. Ves. Pip..

[12] Badgujar D.M., Talawar M.B., Asthana S.N., Mahulikar P.P. (2008). Advances in Science and Technology of Modern Energetic Materials: An Overview. J. Hazard. Mater..

[13] Zhang P., Sun L., Yuan J., Deng J. (2024). Synthesis of Multifunctional Additives for Solid Propellants: Structure, Properties and Mechanism. Def. Technol..

[14] Wu C., Liu Y., Hu S., Lu Y., Guo C., Li H., Qu H., Fu X., Li H. (2023). Correlation between Microstructural Evolution and Mechanical Properties of CMDB Propellant during Uniaxial Tension. Propell. Explos. Pyrot..

[15] Zhang X., Liu Z., Yuan B., Yang K. (2024). Surface Wetting Behaviors of Hydroxyl-Terminated Polybutadiene: Molecular Mechanism and Modulation. Polymers.

[16] Liang J., Nie J., Zhang H., Guo X., Yan S., Han M. (2023). Interaction Mechanism of Composite Propellant Components under Heating Conditions. Polymers.

[17] Zhang X., Deng Z., Xu W., Jiang L., Xu H., Tang Q., Zheng Q., Li J. (2025). Influence of Process Aids on Solid–Liquid Interfacial Properties of Three-Component Hydroxyl-Terminated Polybutadiene Propellants. Polymers.

[18] Liu J., Yu H., Wang D., Sun S., Li F. (2023). Application of Spherical Ultrafine CuO@AP with Core–Shell in AP/HTPB Composite Solid Propellant. J. Therm. Anal. Calorim..

[19] Deng S., Wang J., Sun Y., Luo G., Huan F., Mao C., Wang J. (2025). Effect of Bonding Agent on Interfacial Strength of AP/T313/PBT in PBT-Based Composite Solid Propellants by Molecular Dynamics Simulation. Mater. Today Commun..

[20] Zhang J., Luo P. (2023). Molecular-Level Insights into the Improvement Mechanism of the Bonding Agent MAPO on the Mechanical Properties of the AP/HTPB-TDI Cross-Linked System. Ind. Eng. Chem. Res..

[21] Maimaitituersun W., Wu Y., Hou X., Wang N. (2022). Numerical investigations on mesoscopic structure parameters affecting mechanical responses of propellant. fhclxb.

[22] Lai G.D., Sang L.P., Bian Y.L., Xie H.L., Liu J.H., Chai H.W. (2024). Interfacial Debonding and Cracking in a Solid Propellant Composite under Uniaxial Tension: An In Situ Synchrotron X-Ray Tomography Study. Compos. Sci. Technol..

[23] Kim J., Zhang G., Shi M., Suo Z. (2021). Fracture, Fatigue, and Friction of Polymers in Which Entanglements Greatly Outnumber Cross-Links. Science.

[24] Lian Q., Chen H., Luo Y., Li Y., Cheng J., Liu Y. (2022). Toughening Mechanism Based on the Physical Entanglement of Branched Epoxy Resin in the Non-Phase-Separated Inhomogeneous Crosslinking Network: An Experimental and Molecular Dynamics Simulation Study. Polymer.

[25] Guo Z., Lu X., Wang X., Li X., Li J., Sun J. (2023). Engineering of Chain Rigidity and Hydrogen Bond Cross-Linking toward Ultra-Strong, Healable, Recyclable, and Water-Resistant Elastomers. Adv. Mater..

[26] Cui K., Ma Z., Tian N., Su F., Liu D., Li L. (2018). Multiscale and Multistep Ordering of Flow-Induced Nucleation of Polymers. Chem. Rev..

[27] Wen J.-L., Ming Y.-Q., Zhang A.-F., Li J.-L., Du X.-Y., Shuai L., Nie Y.-J. (2024). Interplay between Hydrogen Bond Network and Entangled Network in Polymers During Stretching Based on Molecular Simulations. Chin. J. Polym. Sci..

[28] Berezkin A.V., Kudryavtsev Y.V. (2015). Effect of Cross-Linking on the Structure and Growth of Polymer Films Prepared by Interfacial Polymerization. Langmuir.

[29] Zhu Y.-L., Liu H., Li Z.-W., Qian H.-J., Milano G., Lu Z.-Y. (2013). GALAMOST: GPU-Accelerated Large-Scale Molecular Simulation Toolkit. J. Comput. Chem..

[30] Martyna G.J., Tobias D.J., Klein M.L. (1994). Constant Pressure Molecular Dynamics Algorithms. J. Chem. Phys..

[31] Sardon H., Irusta L., Fernández-Berridi M.J. (2009). Synthesis of Isophorone Diisocyanate (IPDI) Based Waterborne Polyurethanes: Comparison between Zirconium and Tin Catalysts in the Polymerization Process. Prog. Org. Coat..

[32] Nawaz S., Carbone P. (2014). Coarse-Graining Poly(Ethylene Oxide)–Poly(Propylene Oxide)–Poly(Ethylene Oxide) (PEO–PPO–PEO) Block Copolymers Using the MARTINI Force Field. J. Phys. Chem. B.

[33] Lee H., Choi J.S., Larson R.G. (2011). Molecular Dynamics Studies of the Size and Internal Structure of the PAMAM Dendrimer Grafted with Arginine and Histidine. Macromolecules.

[34] Alessandri R., Grünewald F., Marrink S.J. (2021). The Martini Model in Materials Science. Adv. Mater..

[35] Grunewald F., Rossi G., de Vries A.H., Marrink S.J., Monticelli L. (2018). Transferable MARTINI Model of Poly (Ethylene Oxide). J. Phys. Chem. B.

[36] Milani A., Casalegno M., Castiglioni C., Raos G. (2011). Coarse-Grained Simulations of Model Polymer Nanofibres. Macromol. Theor. Simul..

[37] Giuntoli A., Hansoge N.K., van Beek A., Meng Z., Chen W., Keten S. (2021). Systematic Coarse-Graining of Epoxy Resins with Machine Learning-Informed Energy Renormalization. npj Comput. Mater..

[38] Jacobs R., Morgan D., Attarian S., Meng J., Shen C., Wu Z., Xie C.Y., Yang J.H., Artrith N., Blaiszik B. (2025). A Practical Guide to Machine Learning Interatomic Potentials—Status and Future. Curr. Opin. Solid State Mater. Sci..

[39] Stukowski A. (2009). Visualization and Analysis of Atomistic Simulation Data with OVITO–the Open Visualization Tool. Modelling Simul. Mater. Sci. Eng..

[40] Zou Z., Qiang H., Zhou J., Zhang F., Wang X., Li Y. (2024). Research on Microscopic Structure–Activity Relationship of AP Particle–Matrix Interface in HTPB Propellant. e-Polymers.

[41] Ma L., Zhu X., Zhang W., Zhang H., Wang J., Qu J. (2021). Study on the Preparation and Performance Comparison of Side-Chain Hydroxyl-Terminated Polybutadiene Derivatives with Narrowly Molecular Weight Distribution Used for Polyurethane. Polym. Test..

[42] Liu H., Zhu Y.-L., Lu Z.-Y., Müller-Plathe F. (2016). A Kinetic Chain Growth Algorithm in Coarse-Grained Simulations. J. Comput. Chem..

[43] Yang B., Liu S., Ma J., Yang Y., Li J., Jiang B.-P., Ji S., Shen X.-C. (2022). Monte Carlo Simulation of Surface-Initiated Polymerization: Heterogeneous Reaction Environment. Macromolecules.

[44] Ma J., Li J., Yang B., Liu S., Jiang B.-P., Ji S., Shen X.-C. (2022). A Simple Stochastic Reaction Model for Heterogeneous Polymerizations. Polymers.

[45] Quagliano Amado J.C., Ross P.G., Mattos Silva Murakami L., Narciso Dutra J.C. (2022). Properties of Hydroxyl-Terminal Polybutadiene (HTPB) and Its Use as a Liner and Binder for Composite Propellants: A Review of Recent Advances. Propellants Explos. Pyrotech..

[46] Yoon S., Lee S., Lee J. (2024). Comprehensive Review on Post-Polymerization Modification of Hydroxyl-Terminated Polybutadiene (HTPB). Elastomers Compos..

[47] Nie Y., Gao H., Wu Y., Hu W. (2013). Thermodynamics of Strain-Induced Crystallization of Random Copolymers. Soft Matter.

[48] Wang J., Yu Y., Guo Y., Luo W., Hu W. (2021). Roles of Repeating-Unit Interactions in the Stress Relaxation Process of Bulk Amorphous Polymers. Polymer.

[49] Zheng T., Wang S., Zhou L., Li X., Zhang H. (2022). The Disentanglement and Shear Properties of Amorphous Polyethylene during Friction: Insights from Molecular Dynamics Simulations. Appl. Surf. Sci..

[50] Choi J.B., Nguyen P.C.H., Sen O., Udaykumar H.S., Baek S. (2023). Artificial Intelligence Approaches for Energetic Materials by Design: State of the Art, Challenges, and Future Directions. Propell. Explos. Pyrot..

[51] Han R., Fu X., Qu B., Shi L., Liu Y. (2025). Deep-Neural-Networks-Based Data-Driven Methods for Characterizing the Mechanical Behavior of Hydroxyl-Terminated Polyether Propellants. Polymers.

