# Supplementary material for: Molecular Simulations of Interface-Driven Crosslinked Network Formation and Mechanical Response in Composite Propellants
Source: Polymers (Basel). 2025 Jul 3;17(13):1863. doi: 10.3390/polym17131863 (PMC12251881; doi:10.3390/polym17131863)
Supplement: Supplementary file 1 [file polymers-17-01863-s001.zip › polymers-3719516-supplementary.pdf]

Supplementary Materials for

Molecular Simulations of Interface-Driven Crosslinked Net-work Formation and Mechanical Response in Composite Propellants

Chen Ling [1,#], Xinke Zhang [1,#], Xin Li [2], Guozhu Mou [2], Xiang Guo [2,*], Bing Yuan [3,*] and Kai Yang [1,4*]

1 Center for Soft Condensed Matter Physics and Interdisciplinary Research & School of Physical Science and Technology, Soochow University, Suzhou 215006, Jiangsu, China; 20244208009@stu.suda.edu.cn (C.L.); xkzhang@suda.edu.cn (X.Z.)
2 Hubei Institute of Aerospace Chemical Technology, Xiangyang 441003, Hubei, China
3 Songshan Lake Materials Laboratory, Dongguan 523808, Guangdong, China
4 Jiangsu Key Laboratory of Frontier Material Physics and Devices, Suzhou 215006, Jiangsu, China
# These authors contributed equally to this work.
* Correspondence: yangkai@suda.edu.cn (K.Y.); yuanbing@sslab.org.cn (B.Y.)

Table S1. Setting of LJs, bonds, angles parameters in our model

non-bonded beads		ε	σ
P3	P3	4.9999	0.7
P1	P1	4.4998	0.7
C5	C5	3.5	0.7
C3	C3	3.5	0.7
Q0	Q0	3.5	0.7
IP	IP	4.4998	0.7
HP1	HP1	4.4998	0.7
CR	CR	3.5	0.7
P3	P1	4.4998	0.7
P3	C5	3.5	0.7
P3	C3	3.0998	0.7
P1	C5	3.5	0.7
P1	C3	3.5	0.7
C5	C3	3.5	0.7
P3	Q0	9.9999	0.7
P1	Q0	3.9998	0.7
C5	Q0	3.0998	0.7
C3	Q0	2.2998	0.7
IP	Q0	3.9998	0.7
IP	P3	4.4998	0.7
IP	P1	4.4998	0.7
IP	C5	3.5	0.7
IP	C3	3.5	0.7
HP1	Q0	3.9998	0.7
HP1	P3	4.4998	0.7
HP1	P1	4.4998	0.7
HP1	C5	3.5	0.7
HP1	C3	3.5	0.7
HP1	IP	4.4998	0.7
CR	P3	3.0998	0.7
CR	P1	3.5	0.7
CR	C5	3.5	0.7
CR	C3	3.5	0.7
CR	Q0	2.2998	0.7
CR	IP	3.5	0.7
CR	HP1	3.5	0.7

bonds type	k	l_0
P3-C3	1250	0.4
P3-P1	1250	0.4
P1-C3	1250	0.36
C3-P1	1250	0.36
C3-C3	1250	0.4
C3-C5	1250	0.4
C5-C5	1250	0.36
C5-P1	1250	0.36
C3-IP	1250	0.36
HP1-C3	1250	0.36
SEL	1250	0.4
IPH	1250	0.4
IPM	1250	0.4
IPT	1250	0.4
IPC	1250	0.4
CR-C3	1250	0.4
C3-CR	1250	0.4
CR-CR	1250	0.4

angle type	k_θ	θ_0
C3-P3-C3	25	110
P1-P3-P1	25	110
P1-C3-C3	25	160
C3-C3-P1	25	160
C3-C3-C3	25	140
C3-C5-C5	25	110
C5-C5-P1	25	120
C3-C5-P1	25	120
C3-P1-C5	25	120
P1-C3-P1	25	140
P3-C3-C3	25	160
IP-C3-IP	25	140
HP1-C3-C3	25	160
C3-C3-HP1	25	160
C3-C3-CR	25	140
CR-C3-C3	25	140
C3-CR-C3	25	140
CR-CR-C3	25	140
C3-CR-CR	25	140
CR-C3-CR	25	140
CR-CR-CR	25	140
IPHA	25	160
IPMA	25	160
IPTA	25	160
IPCA	25	160

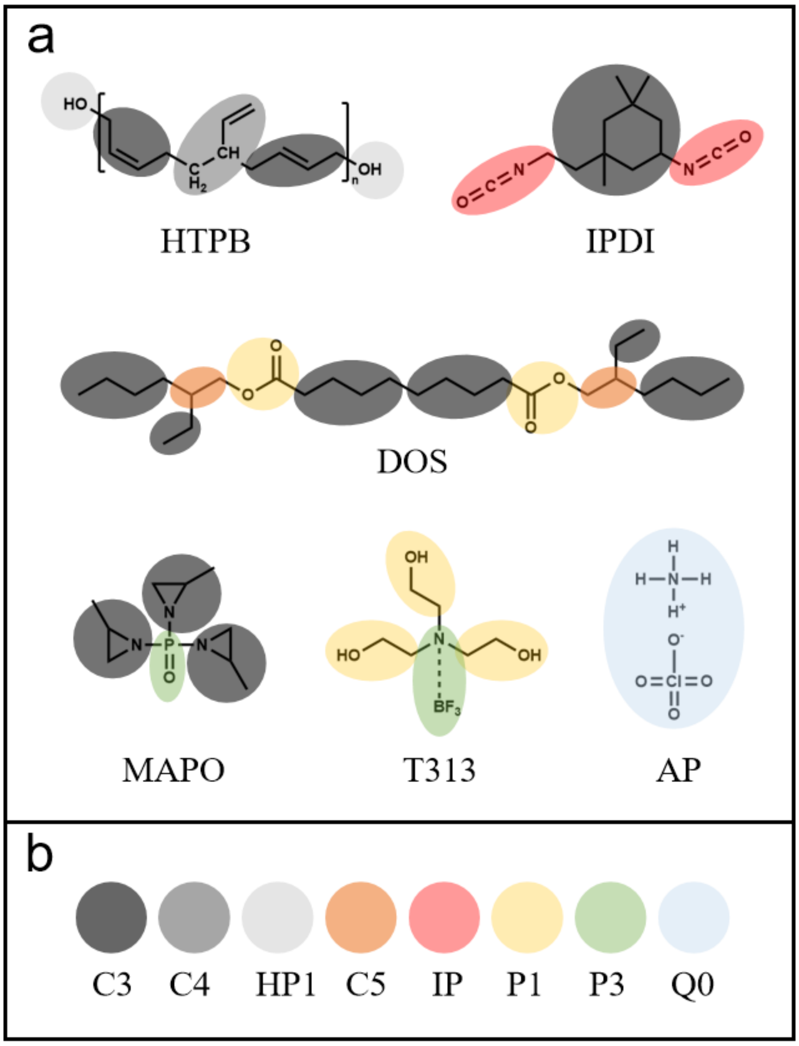

Figure S1. Molecular structures and corresponding coarse-grained modeling scheme.

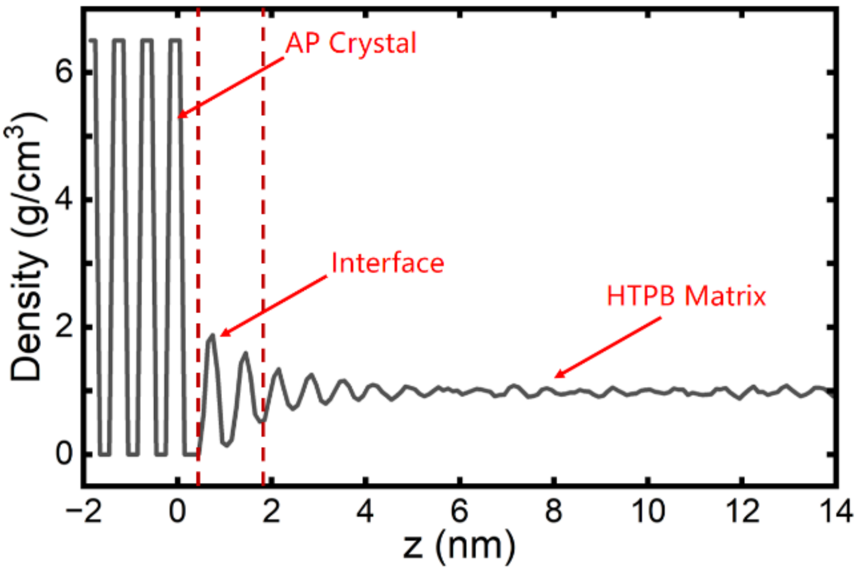

Figure S2. Density distribution of the simulated system, consistent with experimental data and prior all-atom simulations.

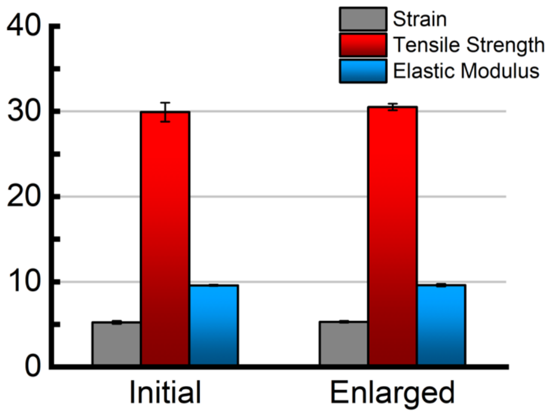

Figure S3. The mechanical properties in initial and scale-up systems.

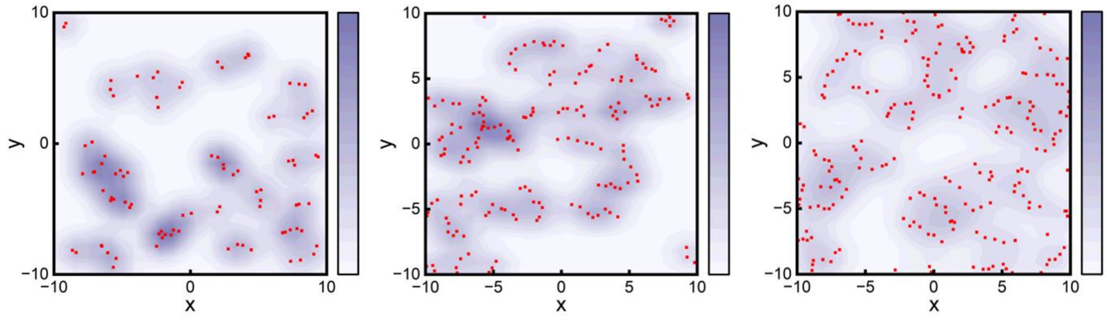

Figure S4. Two-dimensional spatial distribution of MAPO-derived bonds at the AP interface with different MAPO: T313 ratios.

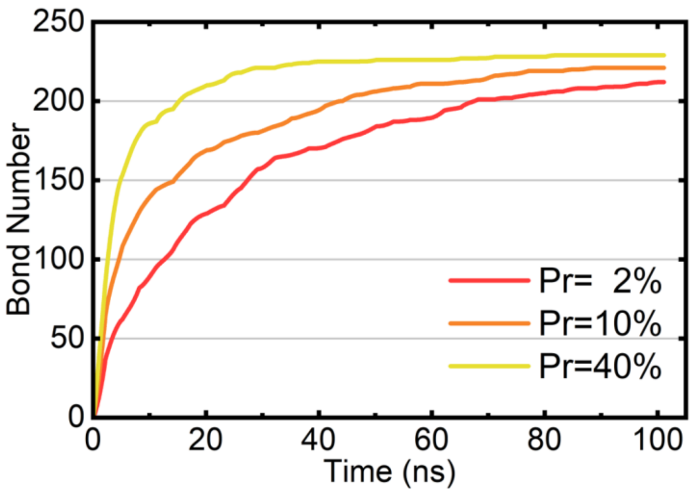

Figure S5. The bond formation process of MAPO self-polymerization with different Pr.

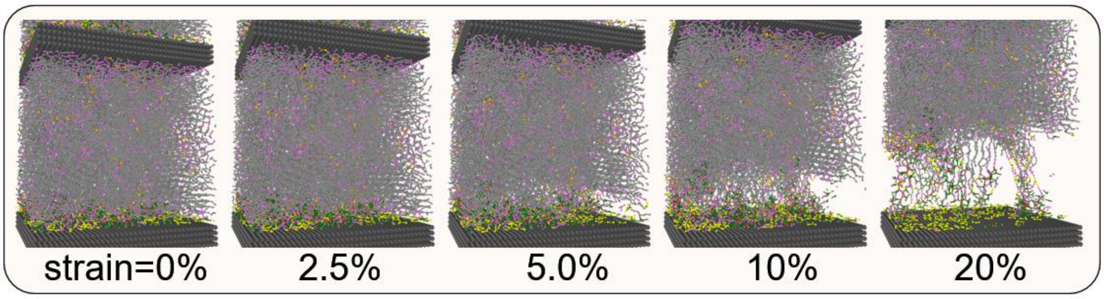

Figure S6. Snapshots of the system under different strains.

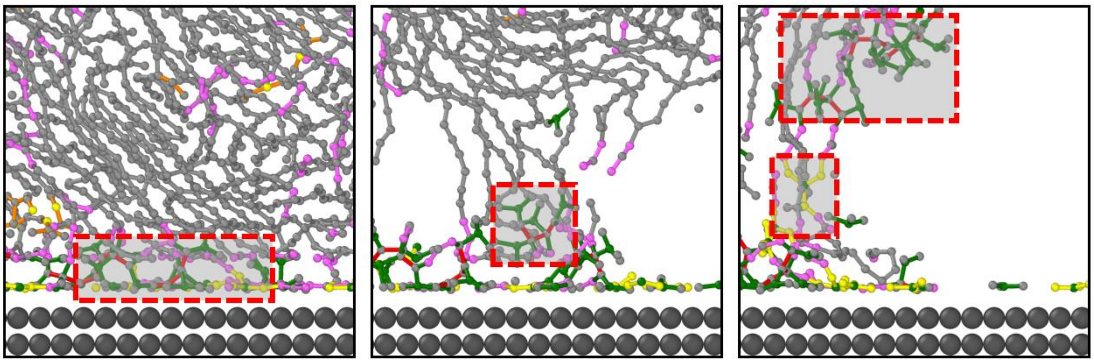

Figure S7. From left to right: (1) the bonding agent is adsorbed onto the AP surface, (2) the bonding agent begins to detach from the interface, and (3) the bonding agent completely departs from the interface.

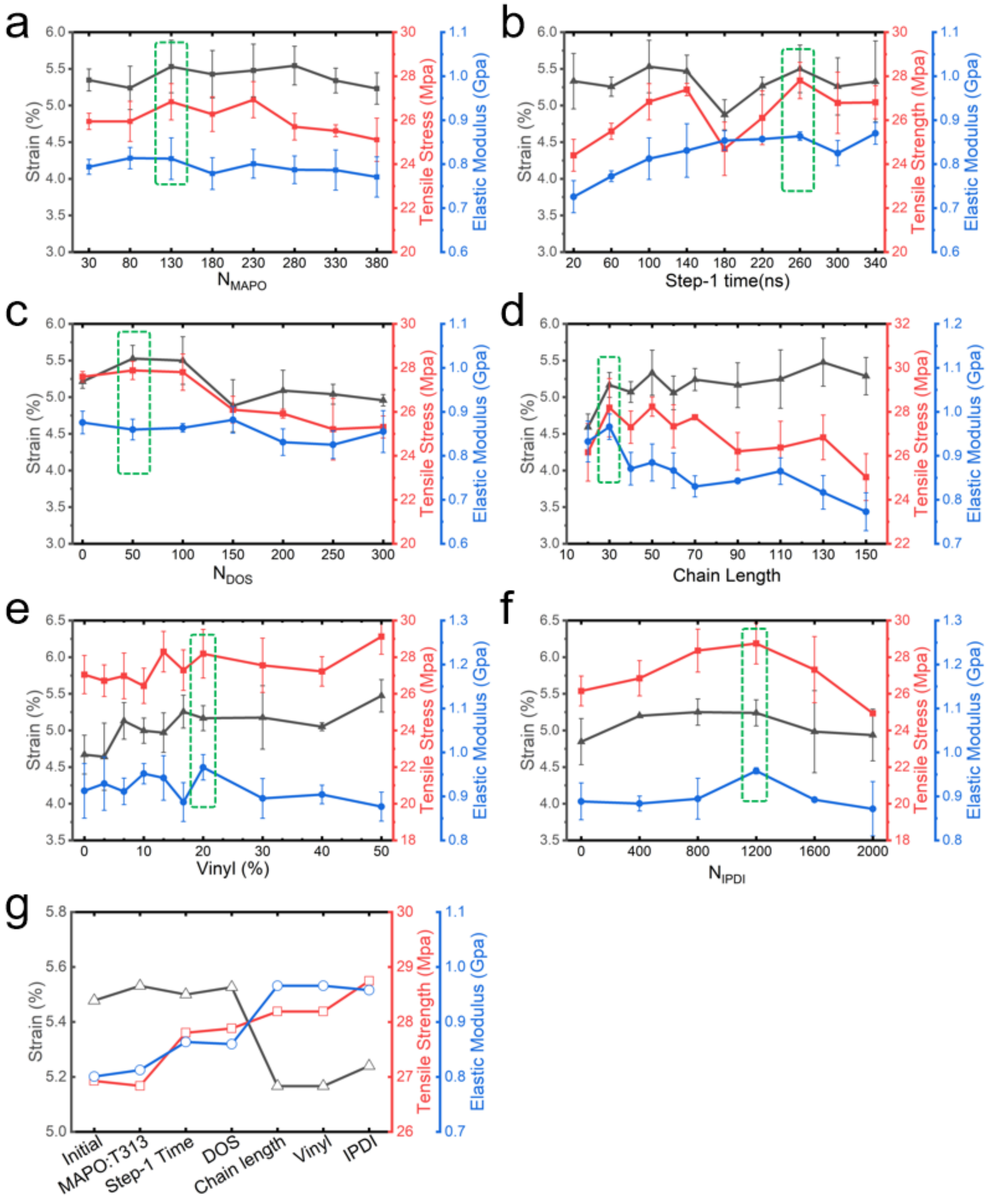

Figure S8. Strain at maximum tensile strength, maximum tensile strength, and elastic modulus as functions of (a) the MAPO: T313 ratio, (b) the duration of the first-step reaction, (c) the amounts of DOS, (d) the HTPB chain length, (e) the proportion of vinyl side chains, and (f) the amount of IPDI. (g) Evolution of mechanical properties during the optimization process. Optimized recipes are outlined in green. For each system, three parallel simulations were performed.
